# Serum amyloid A inhibits astrocyte migration via activating p38 MAPK

**DOI:** 10.1186/s12974-020-01924-z

**Published:** 2020-08-29

**Authors:** Aihua Lin, Jin Liu, Ping Gong, Yanqing Chen, Haibo Zhang, Yan Zhang, Yang Yu

**Affiliations:** 1 Engineering Research Center of Cell and Therapeutic Antibody, Ministry of Education, and School of Pharmacy, Shanghai Jiao Tong University, Shanghai, 200240 China; 2 State Key Laboratory of Oncogenes and Related Genes, Shanghai Cancer Institute, Ren Ji Hospital, School of Medicine, Shanghai Jiao Tong University, Shanghai, 200240 China

**Keywords:** Alzheimer’s disease, Serum amyloid A, Astrocytes, Migration, p38 MAPK

## Abstract

**Background:**

The accumulation of astrocytes around senile plaques is one of the pathological characteristics in Alzheimer’s disease (AD). Serum amyloid A (SAA), known as a major acute-phase protein, colocalizes with senile plaques in AD patients. Here, we demonstrate the role of SAA in astrocyte migration.

**Methods:**

The effects of SAA on astrocyte activation and accumulation around amyloid β (Aβ) deposits were detected in APP/PS1 transgenic mice mated with *Saa3*^*−*/*−*^ mice. SAA expression, astrocyte activation, and colocalization with Aβ deposits were evaluated in mice using immunofluorescence staining and/or Western blotting. The migration of primary cultures of mouse astrocytes and human glioma U251 cells was examined using Boyden chamber assay and scratch-would assay. The actin and microtubule networks, protrusion formation, and Golgi apparatus location in astrocytes were determined using scratch-would assay and immunofluorescence staining.

**Results:**

Saa3 expression was significantly induced in aged APP/PS1 transgenic mouse brain. Saa3 deficiency exacerbated astrocyte activation and increased the number of astrocytes around Aβ deposits in APP/PS1 mice. In vitro studies demonstrated that SAA inhibited the migration of primary cultures of astrocytes and U251 cells. Mechanistic studies showed that SAA inhibited astrocyte polarization and protrusion formation via disrupting actin and microtubule reorganization and Golgi reorientation. Inhibition of the p38 MAPK pathway abolished the suppression of SAA on astrocyte migration and polarization.

**Conclusions:**

These results suggest that increased SAA in the brain of APP/PS1 mice inhibits the migration of astrocytes to amyloid plaques by activating the p38 MAPK pathway.

## Background

Alzheimer’s disease (AD), the major cause of dementia in the elderly, is a chronic progressive neurodegenerative disorder [1]. One of the major neuropathological features of AD is the accumulation of extracellular senile plaques composed of aggregated amyloid β (Aβ). Aggregated Aβ has been proved to play a key role in the occurrence and progression of AD [2, 3]. Besides, neuroinflammation has also been found to contribute to AD [4]. Astrocytes, the most abundant neuroglial cells in the mammalian brain, are involved in AD-related neuroinflammation. Astrocytes are activated in AD and have been found in large numbers to colocalize with and surround Aβ plaques [5, 6]. At the beginning, activated astrocytes that recruit to and accumulate around Aβ plaques are able to take up and degrade Aβ [7]. However, upon sustained activation of Aβ and subsequent stimuli, chronic over-activated astrocytes also secrete pro-inflammatory factors, such as interleukin-1 (IL-1), tumor necrosis factor α (TNF-α), IL-6, and nitric oxide, thereby accelerating the pathological process of AD [8]. These findings suggest that astrocytes exert both protective and detrimental functions in AD. Therefore, the regulation of astrocyte migration and activation might be a promising therapeutic strategy to prevent neurodegeneration.

Serum amyloid A (SAA) is a major acute-phase protein that is released to blood circulation in the event of infection and injury [9]. SAA has cytokine-like properties that regulate several cellular inflammatory responses. SAA induces monocyte and neutrophil migration and stimulates the production and release of cytokines, chemokines, and matrix metalloproteinases (MMPs) [10–16]. In humans, the family of SAA proteins includes SAA1, SAA2, and SAA4 [9]. SAA1 and SAA2 are highly inducible and synthesized by hepatocytes during the acute-phase response. Murine SAA is encoded by a family of three inducible genes, *Saa1*, *Saa2*, and *Saa3*, and a constitutively expressed *Saa4*. Although the inducible isoforms of SAA are synthesized mainly in the liver, they are also produced extrahepatically by a variety of tissues and cells, including macrophages, adipocytes, synovial cells, and tumor cells [17–21]. Murine Saa3 is the predominant SAA isoform induced to be expressed extrahepatically [19, 20, 22–26]. Although the inducible SAA proteins are almost undetectable in normal brains, SAA has been found in the brain of AD patients and to colocalize with Aβ in senile (neuritic) plaques [27, 28]. Moreover, SAA induces the production of inflammatory cytokines including IL-6, TNF-α, IL-12, IL23, and iNOS in primary cultures of mouse astrocytes. SAA inhibits cell proliferation by regulating the cell cycle of astrocytes [29]. All these observations suggest that SAA may contribute to the progression of AD by acting on astrocytes.

In this study, we propose that SAA may play a role in astrocyte migration, thereby affecting the recruitment of astrocytes to Aβ plaques. We first examined the expression of Saa3 in the brain of APP/PS1 transgenic mice at the age of 12 months. Then, we established APP/PS1 transgenic and *Saa3* knockout (APP/PS1-*Saa3*^*−*/*−*^) mice by crossing APP/PS1 transgenic mice with *Saa3*^*−*/*−*^ mice, to investigate the effect of Saa3 on astrocyte activation and migration to Aβ plaques. We found that Saa3 expression was significantly induced in aged APP/PS1 mouse brain. Saa3 deficiency exacerbated astrocyte activation and increased the colocalization of activated astrocytes with Aβ plaques in the cortex and hippocampus of APP/PS1-*Saa3*^*−*/*−*^ mice compared with the APP/PS1 mice, suggesting that the presence of SAA inhibits the migration of astrocytes to Aβ plaques. Furthermore, in vitro studies demonstrated that SAA inhibited astrocyte migration and polarization via disrupting actin and microtubule reorganization and Golgi reorientation. Inhibition of the p38 MAPK pathway abolished the suppression of SAA on astrocyte migration and polarization.

## Methods

### Antibodies and reagents

Dulbecco’s modified Eagle’s medium (DMEM), fetal bovine serum (FBS), and trypsin-ethylenediaminetetraacetic acid (trypsin-EDTA) were purchased from Gibco (Invitrogen, Carlsbad, CA). Lipopolysaccharide (LPS) from *Escherichia coli* 0111:B4 was obtained from Sigma-Aldrich, Inc. (St. Louis, MO). SAA (recombinant human apo-SAA) was purchased from PeproTech (Rocky Hill, NJ). The BCA protein assay kit, 4,6-diamidino-2-phenylindole (DAPI), and FR180204 (ERK inhibitor) were from Beyotime Institute of Biotechnology (Nantong, Jiangsu, China). Rabbit polyclonal anti-Saa3 antibody was from ABclonal Biotechnology Co., Ltd (Wuhan, Hubei, China). Mouse monoclonal anti-GFAP-Cy3™, rabbit polyclonal anti-α-tubulin, and rabbit polyclonal anti-GM130 antibodies were obtained from Sigma-Aldrich, Inc. Rabbit polyclonal anti-Aβ antibody was purchased from Cell Signaling Technology (Danvers, MA). Mouse monoclonal anti-GFAP, mouse monoclonal anti-FITC-phalloidin, and rabbit polyclonal anti-GAPDH antibodies were obtained from Merck KGaA (Darmstadt, Germany); AAT Bioquest, Inc. (Sunnyvale, CA); and Hangzhou Goodhere Biotech Co., Ltd. (Hangzhou, Zhejiang, China), respectively. Mouse and rabbit control IgGs were purchased from Santa Cruz Biotechnology (Dallas, TX). Alexa Fluor 488-conjugated anti-rabbit IgG secondary antibody was from Gibco. IRDye® 800CW and IMDye® 800CW secondary antibodies were from LI-COR, Inc. (Lincoln, NE). Other reagents were obtained from Sigma-Aldrich.

### Animals

The APP/PS1 transgenic mice in C57BL/6J background (APP_SWE_/PS1ΔE9^+/−^, stock number 005864) were purchased from The Jackson Laboratory (Bar Harbor, ME). The *Saa3* knockout (*Saa3*^*−*/*−*^) mice in C57BL/6J background were obtained from the Knockout Mouse Project (KOMP) Repository (Davis, CA). *Saa3*^*−*/*−*^ mice were crossed to APP/PS1 mice to generate APP/PS1-*Saa3*^+/−^ mice, and then the latter are further crossed with *Saa3*^+/−^ mice to create the following four groups: WT (APP/PS1^*−*/*−*^-*Saa3*^+/+^), APP/PS1 (APP/PS1^+/−^-*Saa3*^+/+^), *Saa3*^*−*/*−*^ (APP/PS1^*−*/*−*^-*Saa3*^*−*/*−*^), and APP/PS1-*Saa3*^*−*/*−*^ (APP/PS1^+/−^-*Saa3*^*−*/*−*^). Mouse genotypes were determined by PCR. All mice were housed (4–5 animals per cage) with a 12/12-h light/dark cycle, with ad libitum access to food and water. The housing, breeding, and animal experiments were in accordance with the National Institutes of Health Guide for the Care and Use of Laboratory Animals, with procedures approved by the Biological Research Ethics Committee, Shanghai Jiao Tong University. All the four groups of mice at the age of 12 months were sacrificed by decapitation, and their brains were removed immediately. The cerebral cortices and hippocampi of the left hemisphere of the brain were dissected, flash-frozen in dry ice, and stored at − 80 °C for Western blot later. The right hemispheres of the brain were fixed with 4% paraformaldehyde in 0.1 M phosphate-buffered saline (PBS), followed by cryoprotection in 30% sucrose. Sagittal sections of 30-μm thickness were cut using a freezing sliding microtome. The sections were stored in glycol anti-freeze solution (12.5 g/L polyvinylpyrrolidone (average MW 40,000), 375 g/L saccharose, 375 mL/L glycol, 625 mL/L Tris-buffered saline (TBS, 0.1 M, containing 12.1 g/L Tris-base, 40 g/L NaCl)) at − 20 °C until immunofluorescence staining.

### Primary astrocyte culture

Astrocyte cultures were prepared from 1-day-old C57BL/6J WT mouse pups as previously described [26]. Briefly, the cerebral cortices were removed from the brains of mice, then the meanings and microvessels were removed. Tissues were minced with a sterile ophthalmic scissor and digested with 0.05% trypsin at 37 °C for 10 min. The cell suspension was filtered through a 40-μm sieve, then cells were plated on poly-d-lysine-coated 75-cm^2^ flasks with DMEM (containing 10% FBS, 100 U/mL penicillin, and 100 μg/mL streptomycin sulfate). The medium was replenished on day 1 and day 3. On day 7, the microglia in the culture flasks were shaken off at 260 rpm for 2.5 h, and the remaining astrocytes were maintained in DMEM with 10% FBS. Experiments were performed after one passage of the cells.

Human glioma U251 cells were kindly provided by Dr. Zejian Wang (Shanghai Jiao Tong University, China). U251 cells were grown in DMEM supplemented with 10% FBS, 2 mM glutamine, 100 U/mL penicillin, and 100 μg/mL streptomycin sulfate.

### Western blot

Mouse brain tissue was homogenized in lysis buffer containing 50 mM Tris-HCl (pH 7.4), 100 mM NaF, 2 mM EDTA, 10 mM β-mercaptoethanol, 2 mM NaVanadate, 8.5% sucrose, 5 μg/mL aprotinin, 100 μg/mL leupeptin, and 5 μg/mL pepstatin. Protein concentrations were determined using a BCA Kit according to the manufacturer’s protocol. Then, the tissue homogenates were heated in 5× sodium dodecyl sulfate (SDS)-PAGE loading buffer at 99 °C for 10 min. Tissue homogenates were separated on 10% SDS-PAGE, and separated samples were transferred onto nitrocellulose membranes (Whatman Protran®, Sigma). The membranes were blocked with 5% non-fat milk for 1 h at room temperature and incubated overnight at 4 °C with primary antibodies including anti-GFAP (1:1000) and anti-GAPDH (1:1000), followed with corresponding secondary antibodies IRDye® 800CW or IMDye® 800CW antibodies for 1 h at room temperature. The membranes were scanned using the 800-nm channel of an Odyssey® P140-CLx Infrared Imaging System (LI-COR, Inc.). Densitometric quantification of protein bands was analyzed using the ImageJ software (National Institute of Mental Health, Bethesda, MD).

### Immunofluorescence staining

Sections of mouse brain or cultured cells were processed for standard immunofluorescence staining [30]. Briefly, sections or cells were washed with 0.05 M TBS for three times at room temperature, permeabilized with 0.1% Triton X-100 in TBS for 10 min, and blocked with 5% normal goat serum in TBS (0.1% Tween-20) for an additional 30 min. Then, the samples were incubated overnight at 4 °C with anti-Saa3 (1:200) or anti-Aβ (1:200) in TBS. After rinsing with TBS for three times, the samples were incubated with Alexa Fluor 488-conjugated anti-rabbit secondary antibody (1:500) at room temperature for 1 h. For double immunofluorescence staining of Aβ and GFAP, after staining of Aβ, the sections were further incubated with anti-GFAP-Cy3™ (1:500) for another 1 h. After 30-min washes in TBS, the samples were stained for nuclei with 100 ng/mL of DAPI for 10 min and mounted with 90% glycerol. The fluorescent confocal images were taken on a laser scanning confocal fluorescent microscope (TCS SP8, Leica Microsystems, Wetzlar, Germany). For quantification of the expression of Saa3 and GFAP, the immunofluorescence intensity was quantified using the ImagePro Plus Software (Media Cybernetics, Silver Spring, MD). Data are presented as the mean ± SEM based on four individual fields for each region, using at least two mice in each group, or from at least three independent experiments, each in triplicate.

### Boyden chamber assay

In this study, standard 48-well chemotaxis chambers (Neuro Probe, Gaithersburg, MD), in which the upper and lower wells were separated by a polycarbonate membrane (8-μm pore size), were used to study the motility of astrocytes [30, 31]. The lower wells of the chamber were added with either control DMEM or DMEM containing various concentrations of SAA. The membrane was an 8-μm pore size polycarbonate filter (Neuro Probe) over the lower wells of the chamber. The upper chamber wells were filled with primary cultures of astrocytes or U251 cells with or without a 15-min pre-treatment with SB203580 (10 μM), FR180204 (5 μM), SP600125 (5 μM), or LY294002 (5 μM). After primary astrocytes or U251 cells were added to the upper wells, the chamber was incubated in a humidified incubator with 5% CO_2_. After incubation at 37 °C for 12 h, the filter was carefully removed. Astrocytes on the upper surface of the filter that did not migrate were wiped off with a cotton-tipped swab. The remaining astrocytes that migrated to the bottom surface of the filter were fixed with methanol, stained with 0.1% crystal violet, and counted with a phase contrast inverted microscope (IX51, Olympus Optical Co. Ltd., Tokyo, Japan), custom-fitted with a digital camera (EOS 1100D, Canon Inc., Tokyo, Japan). The results were expressed as the chemotaxis index which represents the fold increase or decrease in the number of migrated cells in response to chemoattractant over that of control medium. Data are presented as the mean ± SEM from three independent experiments, each with three wells for each group.

### Scratch-would assay

The scratch-would assay was performed as described previously [32, 33]. A scratch was made in confluent primary astrocytes and U251 cells grown on poly-l-ornithine-precoated coverslips using a 10-μL pipette tip, then a 500-μm-wide cell-free area was generated. The movement images of primary astrocytes and U251 cells were taken under light microscopy (Olympus Optical Co. Ltd.). Pictures were captured at 0 h as control. Then, the cells were incubated with SAA (1 μM) with or without a 15-min pre-treatment with SB203580 (10 μM), FR180204 (5 μM), SP600125 (5 μM), or LY294002 (5 μM) for 8 h, 12 h, or 16 h, and the pictures were captured at each of the time points. The results were quantified by calculating the mean migrated distance of leading cells in the scratched area.

To detect actin and microtubule networks, the immunofluorescence staining was performed as described above. The cells were stained with anti-FITC-phalloidin (1:1000) for F-actin and anti-α-tubulin (1:1000) for microtubule at 16 h after scratching. The secondary antibody Alexa Fluor 488-conjugated anti-rabbit antibody (1:500) against the anti-α-tubulin was used.

### Protrusion formation

Protrusion formation was examined at 16 h after scratching following fixation and microtubule staining [34]. The primary cultures of astrocytes and U251 cells with staining were scored as protruding when their protrusions were at least 4 times longer than wide.

### Golgi reorientation

The cells were fixed and stained with anti-GM130 antibody (1:2000) at 12 h after scratching. The secondary antibody Alexa Fluor 488-conjugated anti-rabbit antibody (1:500) against the anti-GM130 antibody was used. The immunofluorescence staining was performed as described above. The location of Golgi in front of the nucleus, within 120° sectors facing the wound, was considered positive.

### Statistical analyses

Data are presented as means ± SEM. Two-group comparisons were evaluated by the two-tailed *t* test. Multiple comparisons were analyzed using one-way ANOVA, followed by Tukey’s post hoc test. All analyses were performed with the statistical software GraphPad Prism 8 (San Diego, CA). *p* < 0.05 was considered statistically significant.

## Results

### Elevated expression of Saa3 in the brain of APP/PS1 mice

**Fig. 1:**
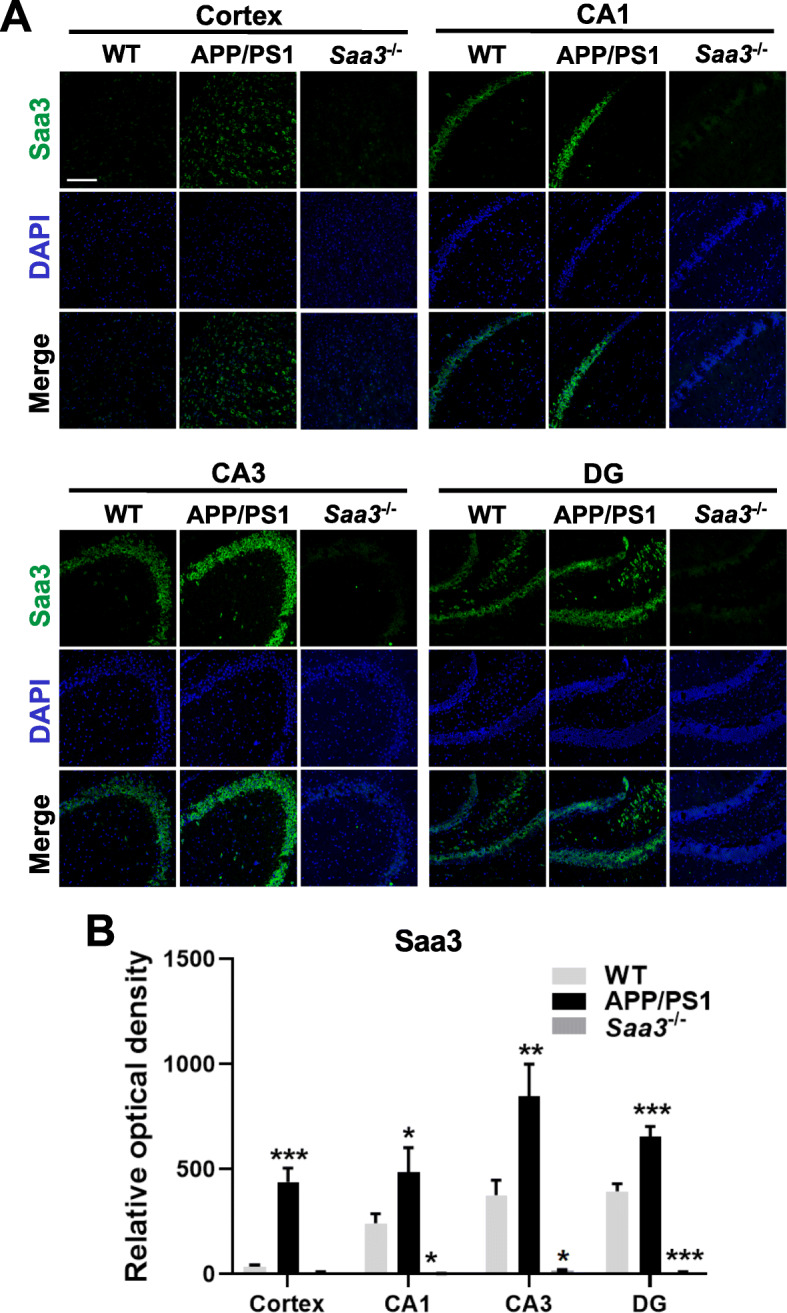
Upregulation of Saa3 in the cortex and hippocampus of APP/PS1 transgenic mice. **a** Serial sections of 12-month-old WT, APP/PS1, and *Saa3*^*−*/*−*^ mouse brain were stained for Saa3 protein using a rabbit anti-Saa3 polyclonal antibody and Alexa Fluor 488-conjugated anti-rabbit IgG (*green fluorescence*). The cell nuclei were stained with DAPI (*blue*). The scale bar in the upper left panel is 150 μm. **b** Quantification of the Saa3 fluorescence was shown. Results are expressed as the mean ± SEM based on four individual fields for each region, using 3–4 mice in each group. *p < 0.05; **p < 0.01; ***p < 0.001 compared with WT mice

To identify the potential role for SAA in AD, we first investigated the expression of SAA in APP/PS1 mouse brain. Immunofluorescence staining of serial slices from mouse brain with an anti-Saa3 antibody identified a significantly elevated expression of Saa3 (green fluorescence) in the cortex as well as in the cornu ammonis (CA) and dentate gyrus (DG) regions of the hippocampus of APP/PS1 mice aged 12 months, compared with the WT mice of the same age (Fig. 1a, b). In addition, it is obvious that increased Saa3 was mainly colocalized with neurons based on the patterns of staining (Fig. 1a). As expected, no obvious fluorescent staining of Saa3 was observed in the brain of *Saa3*^*−*/*−*^ mice. These findings led us to examine the possible role of SAA in the pathological process of AD.

### Saa3 deficiency increases astrocyte activation and colocalization with Aβ plaques in APP/PS1 mice

**Fig. 2:**
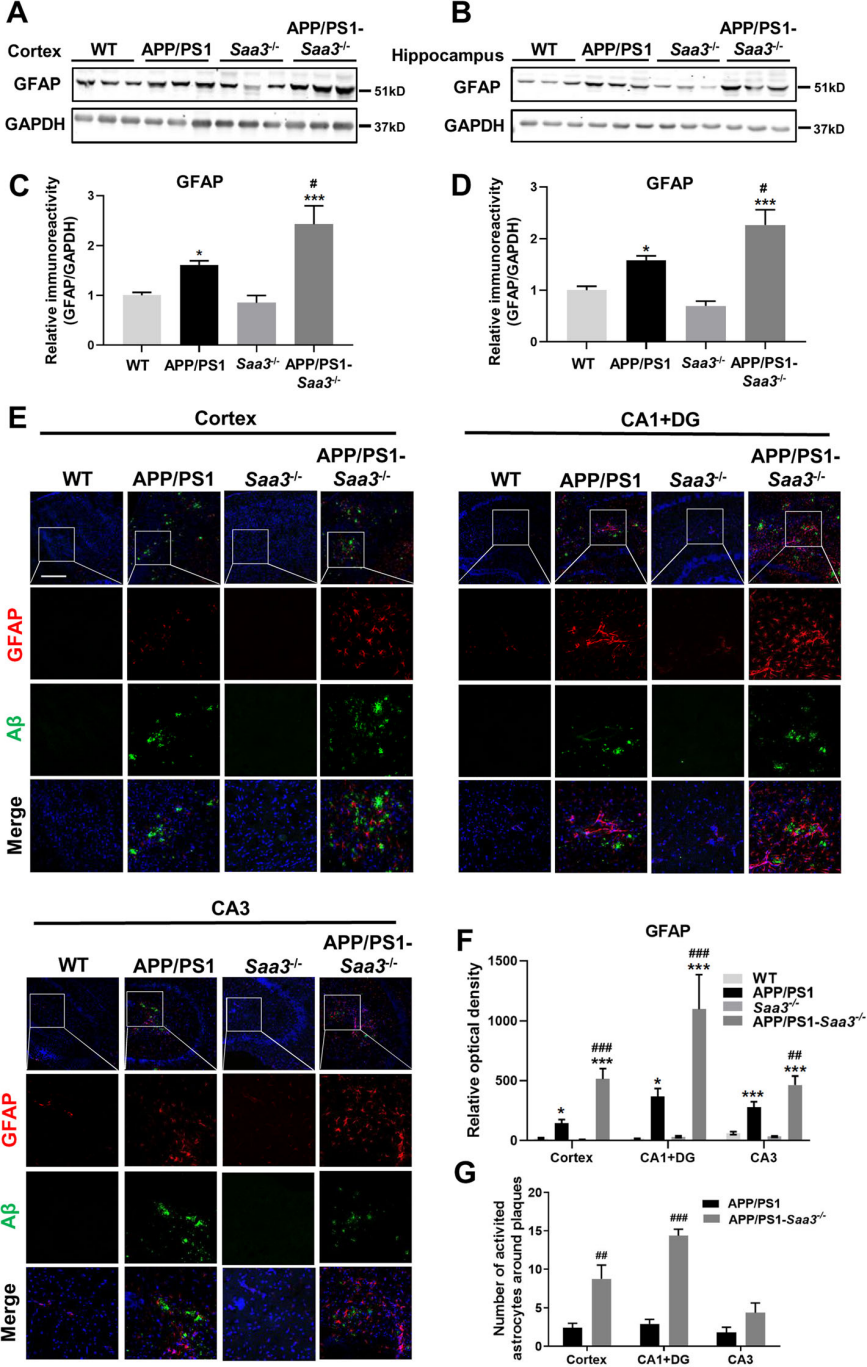
Saa3 deficiency increases the number of activated astrocytes around Aβ plaques in the brain of APP/PS1 mice. Representative Western blots showing the GFAP expression in the cortex (**a**) and hippocampus (**b**) of 12-month-old WT, APP/PS1, *Saa3*^*−*/*−*^, and APP/PS1-*Saa3*^*−*/*−*^ mice. Quantification of the immunoreactivity of the blots normalized against GAPDH was shown in **c** and **d**. Results are expressed as the mean ± SEM, with 3–8 mice in each group. *p < 0.05; ***p < 0.001 compared with WT mice. ^#^*p* < 0.05 compared with APP/PS1 mice. **e** Serial sections of 12-month-old WT, APP/PS1, *Saa3*^*−*/*−*^, and APP/PS1-*Saa3*^*−*/*−*^ mouse brain were stained for Aβ deposits using a rabbit anti-Aβ polyclonal antibody and Alexa Fluor 488-conjugated anti-rabbit IgG (*green fluorescence*). The sections were subsequently stained for GFAP using a monoclonal anti-GFAP Cy3^TM^ antibody (*red fluorescence*). The cell nuclei were stained with DAPI (*blue*). The scale bar in the upper left panel is 300 μm. Selected areas are enlarged by seven times and shown as combined as well as individual fluorescence stains. Quantification of the GFAP fluorescence (**f**) and the number of astrocytes around Aβ deposits (**g**) was shown. Results are expressed as the mean ± SEM based on four individual fields for each region, using 2–3 mice in each group. *p < 0.05; ***p < 0.001 compared with WT mice. ^##^*p* < 0.01; ^###^*p* < 0.001 compared with APP/PS1 mice

Since SAA plays a role in inflammatory response by acting on glial cells [29] and its expression is significantly induced in AD mouse brain, we next explored whether SAA is involved in astrocyte activation and migration toward Aβ deposits in AD mice. We generated APP/PS1-*Saa3*^*−*/*−*^ mice and investigated the expression of the glial fibrillary acidic protein (GFAP), a marker of reactive astrocytes, and its colocalization with Aβ deposits in these mice. We crossed APP/PS1 transgenic mice on a C57BL/6 background to *Saa3*^*−*/*−*^ mice (also on a C57BL/6 background) to generate APP/PS1-*Saa3*^+/−^ mice, then crossed APP/PS1-*Saa3*^+/−^ mice with *Saa3*^+/−^ mice to generate APP/PS1-*Saa3*^*−*/*−*^ mice. We analyzed the brains of APP/PS1-*Saa3*^*−*/*−*^ mice at the age of 12 months in comparison with APP/PS1 mice of the same age. The level of GFAP was determined by Western blot. As shown in Fig. 2a–d, GFAP expression was significantly increased in the cortex and hippocampus of APP/PS1 mice compared with WT mice. Saa3 deficiency induced a further increase of GFAP expression in APP/PS1-*Saa3*^*−*/*−*^ mice compared with APP/PS1 mice (Fig. 2a–d). In addition to Western blot, immunofluorescence staining was also performed. We stained frozen slices of brain tissue with CY3™-conjugated mouse monoclonal anti-GFAP (red fluorescence). As shown in Fig. 2e, increased red fluorescence was observed in the cortex and in the CA1, DG, and CA3 regions of the hippocampus in APP/PS1 mice compared with WT mice. The red fluorescence was further enhanced in activated astrocytes with enlarged cell bodies and elongated and thickened protrusions in the brain of APP/PS1-*Saa3*^*−*/*−*^ mice compared with APP/PS1 mice (Fig. 2e, f). In addition, Western blot and immunofluorescence assay showed no obvious astrocyte activation in WT and *Saa3*^*−*/*−*^ mouse brain. All these results suggest that the absence of SAA further enhances the activation of astrocytes in the brain of AD mice.

Next, to explore whether SAA is involved in astrocyte migration toward Aβ plaques, double immunofluorescence staining was conducted for GFAP (red fluorescence) and Aβ deposits (green fluorescence). As shown in Fig. 2e, g, immunofluorescence staining and quantitative analysis revealed a significant increase in the number of activated astrocytes colocalized with Aβ deposits in the cortex and in the CA1 and DG regions of the hippocampus of APP/PS1-*Saa3*^*−*/*−*^ mice, compared with the APP/PS1 mice. In the CA3 region, astrocytes colocalized with Aβ plaques also tended to increase, although not statistically significant (Fig. 2g). These results suggest that the presence of SAA may inhibit the migration of astrocytes to Aβ plaques.

### SAA inhibits the migration of primary astrocytes and U251 cells

**Fig. 3:**
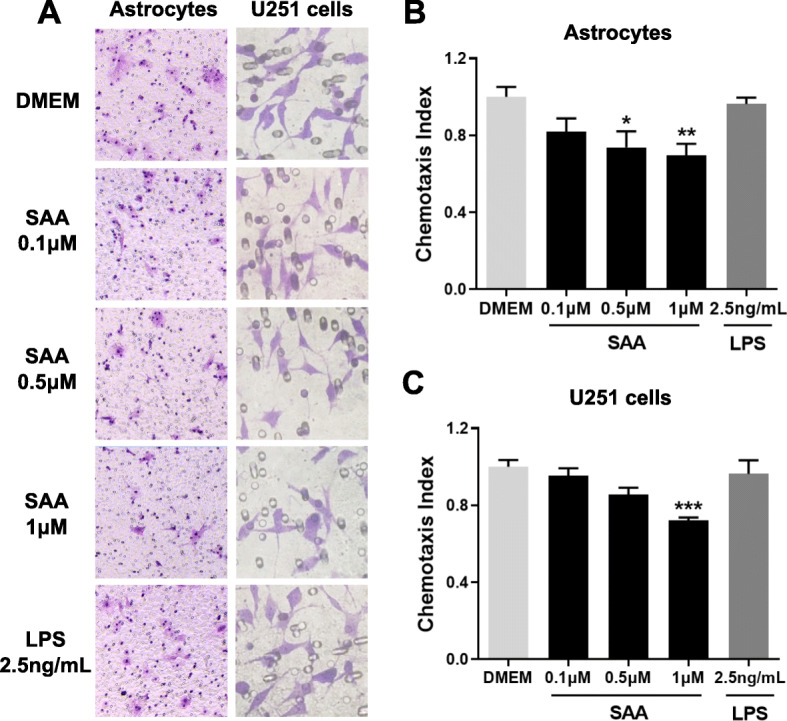
SAA inhibits astrocyte migration in Boyden chamber assay. Primary cultures of astrocytes and U251 cells were treated for 12 h with SAA (0.1–1 μM) or LPS (2.5 ng/mL). The cell migration was examined using 48-well chemotaxis chambers. Representative images of migrated cells (primary astrocytes and U251 cells) on membrane filters were shown in **a**, and quantified data were shown in **b** and **c**. Magnification, × 400. Results are expressed as the mean ± SEM based on three independent experiments, each with three wells for each group. *p < 0.05; **p < 0.01; ***p < 0.001 compared with the control (DMEM)

To verify the role of SAA in astrocyte migration, the Boyden chamber assay was performed. SAA (0.1–1 μM) as the chemoattractant was applied to the lower chamber wells, and primary cultures of mouse astrocytes or human glioma U251 cells were added to the upper wells. The cells that migrated across the filter over 12 h were counted. The results showed that SAA in the lower chamber inhibited the migration of primary astrocytes and U251 cells from the upper chamber through the filter to the bottom surface of the filter, in a dose-dependent manner (Fig. 3a–c). To exclude the influence of trace LPS contamination in recombinant human apo-SAA (1.14 ng/mL LPS for 1 μM SAA), we tested 2.5 ng/mL LPS on astrocyte migration and found no significant effect on astrocyte migration at the concentration (2.5 ng/mL) and time (12 h) tested (Fig. 3a–c). In addition, MTT assay showed that SAA (1 μM) treatment for 16 h had no effect on the viability of primary astrocytes and U251 cells (Additional file 1: Figure S1A).

**Fig. 4:**
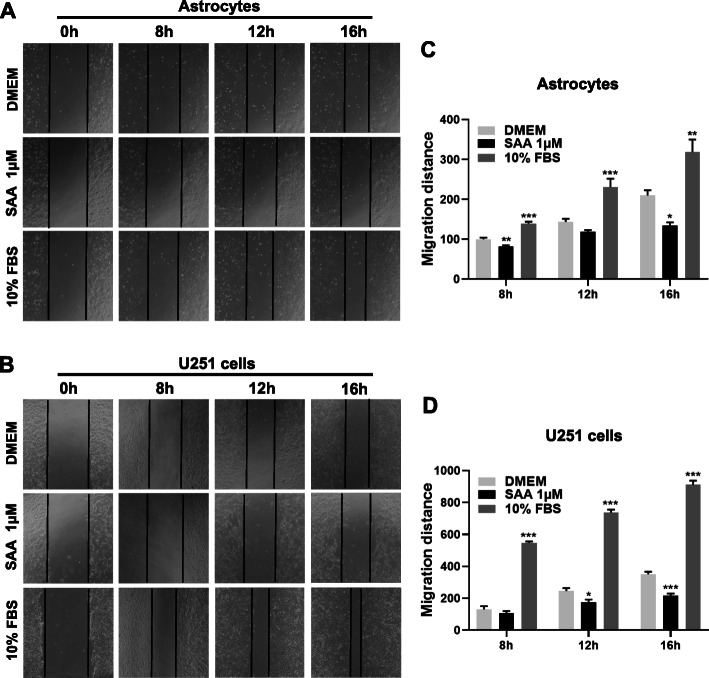
SAA inhibits astrocyte migration in scratch-wound assay. Primary cultures of astrocytes and U251 cells were treated with SAA (1 μM) or 10% FBS. The cell migration was examined using scratch-wound assay. The cells were photographed at 0 h, 8 h, 12 h, and 16 h. Representative images of migrated cells (primary astrocytes and U251 cells) were shown in **a** and **b**, and quantified data were shown in **c** and **d**, respectively. Magnification, × 100. Results are expressed as the mean ± SEM based on three independent experiments, each in triplicate. *p < 0.05; **p < 0.01; ***p < 0.001 compared with the control (DMEM)

In addition to the Boyden chamber assay, scratch-wound assay was also conducted to investigate the inhibition effect of SAA on astrocyte migration. For the scratch-wound assay, primary cultures of astrocytes and U251 cells were treated with 1 μM SAA or 10% FBS for 8 h, 12 h, and 16 h. Images were taken, and wound closure was quantified after 8 h, 12 h, and 16 h. Consistent with the result of the Boyden chamber assay, a significant inhibition of wound closure was found in primary astrocytes and U251 cells treated with SAA compared with cells treated with DMEM alone (Fig. 4a–d). As a control, 10% FBS treatment significantly promoted wound closure of primary astrocytes and U251 cells (Fig. 4a–d). All these results suggest that SAA inhibits astrocyte migration.

### SAA inhibits actin and microtubule reorganization and Golgi reorientation in astrocytes

**Fig. 5:**
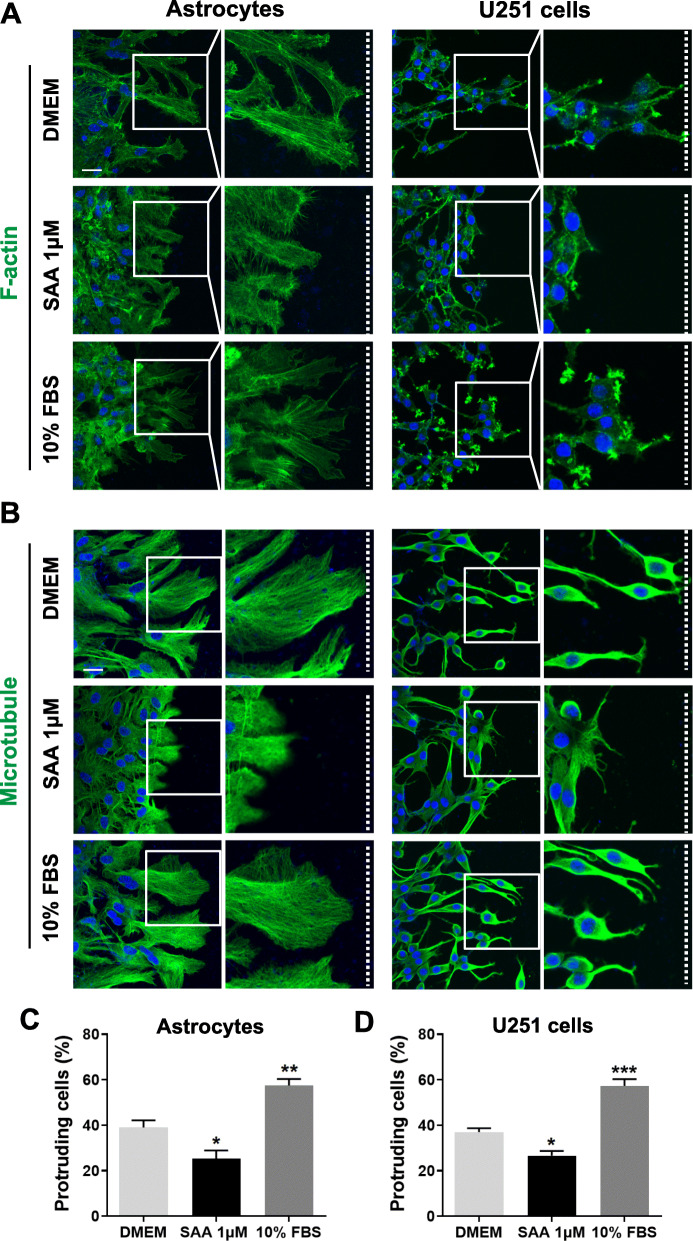
SAA inhibits actin and microtubule reorganization in astrocytes with scratch-wound assay. Primary cultures of astrocytes and U251 cells were treated with SAA (1 μM) or 10% FBS for scratch-wound assay. At 16 h after scratching, the cells were stained with anti-FITC-phalloidin (F-actin, *green fluorescence*) (**a**) or anti-α-tubulin (microtubule) polyclonal antibody and Alexa Fluor 488-conjugated anti-rabbit IgG (*green fluorescence*) (**b**). The cell nuclei were stained with DAPI (*blue*). Scale bar, 25 μm. The white dashed lines indicate the direction of the wound. Selected areas are enlarged by four times. For microtubule staining, primary astrocytes (**c**) and U251 cells (**d**) were scored as protruding when their protrusions were at least 4 times longer than wide. Results are expressed as the mean ± SEM based on three independent experiments, each in triplicate. *p < 0.05; **p < 0.01; ***p < 0.001 compared with the control (DMEM)

Since cell polarization is a requirement of cellular migration and movement [35], we further investigated whether SAA affects the polarization of astrocytes. For scratch-wound assay, a scratch was made in confluent primary cultures of astrocytes and U251 cells, which were treated with 1 μM SAA or 10% FBS. Then, the cells were stained with anti-phalloidin (F-actin) or anti-α-tubulin at 16 h after scratching. In the control astrocyte cultures, the scratch induced a significantly polarized morphology of cells at the wound edge (Fig. 5a, b), which is characterized by the formation of long protrusions filled with elongated actin and microtubule networks arranged parallel to the protrusion axis. However, SAA treatment attenuated the formation of protrusion in wound-edge cells (Fig. 5a, b). The astrocytes showed short protrusions and disorganized actin and microtubule networks (Fig. 5a, b). In addition, for microtubule staining, quantitative analysis showed a significant decrease in the number of protruding cells at the wound edge in primary astrocytes and U251 cells treated with SAA compared with cells treated with DMEM alone (Fig. 5c, d). These results suggest that SAA inhibits the polarization of astrocytes by disrupting the reorganization of actin and microtubule networks in the protrusions.

**Fig. 6:**
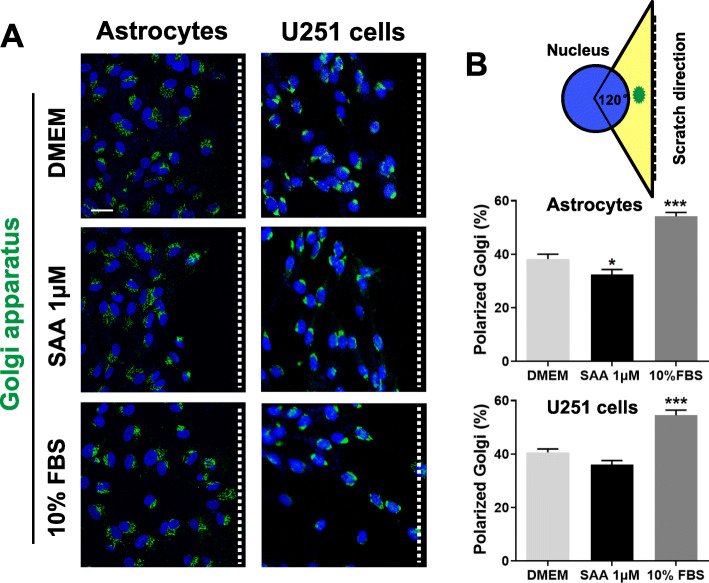
SAA inhibits Golgi apparatus reorientation in astrocytes with scratch-wound assay. **a** Primary cultures of astrocytes and U251 cells were treated with SAA (1 μM) or 10% FBS for scratch-wound assay. At 12 h after scratching, the cells were stained with anti-GM130 (Golgi apparatus) polyclonal antibody and Alexa Fluor 488-conjugated anti-rabbit IgG (*green fluorescence*). The cell nuclei were stained with DAPI (*blue*). Scale bar, 25 μm. The white dashed lines indicate the direction of the wound. **b** The percentage of primary astrocytes and U251 cells with the Golgi in the forward-facing 120° sectors (yellow on the diagram) was measured in wound-edge cells. Results are expressed as the mean ± SEM based on three independent experiments, each in triplicate. *p < 0.05; ***p < 0.001 compared with the control (DMEM)

Next, we investigated the effect of SAA on the orientation of the Golgi apparatus in the scratch-wound assay, since the Golgi apparatus is another requirement in cell polarization and directed migration. We detected the reorientation of Golgi in wound-edge cells to the direction of migration at 12 h after scratching. In the control astrocyte cultures, the Golgi in about 40% of the wound-edge cells oriented toward the direction of the scratch (Fig. 6). SAA treatment reduced the reorientation of Golgi toward the wound leading edge in primary astrocytes and U251 cells compared with cells treated with DMEM alone (Fig. 6). As expected, 10% FBS induced a pronounced reorientation of Golgi toward the scratch in cells compared with cells treated with DMEM alone (Fig. 6). All these data suggest that SAA inhibits astrocyte migration and polarization via disrupting actin and microtubule reorganization and Golgi reorientation.

### Inhibiting p38 MAPK attenuates the interference of SAA on astrocyte migration and polarization

**Fig. 7:**
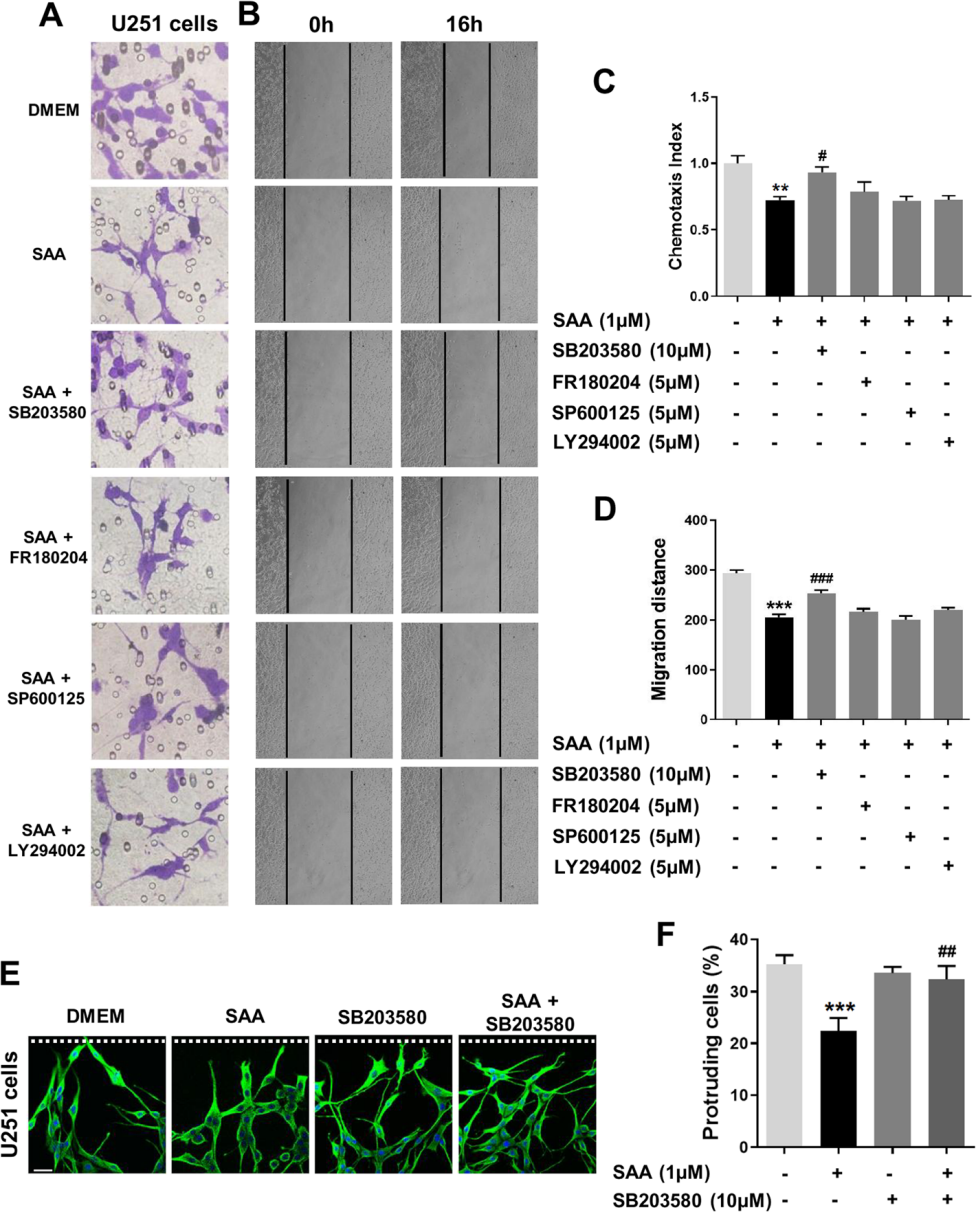
Inhibiting p38 alleviates the interference of SAA on astrocyte migration and polarization. U251 cells were incubated with SAA (1 μM) with or without a 15-min pre-treatment with SB203580 (10 μM), FR180204 (5 μM), SP600125 (5 μM), or LY294002 (5 μM). At 12 h and 16 h after incubation, the cell migration was examined by 48-well chemotaxis chambers (**a**) and scratch-wound assay (**b**), and the quantified data were shown in **c** and **d**. Magnification, × 400 and × 100, respectively. Results are expressed as the mean ± SEM based on three independent experiments, each with three wells for each group or in triplicate. **p < 0.01; ***p < 0.001 compared with the control (DMEM). ^#^*p* < 0.05; ^###^*p* < 0.001 compared with SAA treatment group. **e** To detect protrusion formation, U251 cells were incubated with SAA (1 μM) with or without a 3-h pre-treatment with SB203580 (10 μM). At 16 h after scratching, the cells were stained with anti-α-tubulin (microtubule) polyclonal antibody and Alexa Fluor 488-conjugated anti-rabbit IgG (*green fluorescence*). The cell nuclei were stained with DAPI (*blue*). Scale bar, 25 μm. The white dashed lines indicate the direction of the wound. The cells were scored as protruding when their protrusions were at least 4 times longer than wide. **f** The quantified data were shown. Results are expressed as the mean ± SEM based on three independent experiments, each in triplicate. ***p < 0.001 compared with the control (DMEM). ^##^*p* < 0.01 compared with SAA treatment group

Studies have demonstrated that MAPKs and PI3K play crucial roles in cell migration [36, 37]. Next, we explored whether the inhibition of SAA on astrocyte migration and polarization is related to these kinases. Our previous study has found that SAA induces the activation of the p38, ERK1/2, JNK, and PI3K signaling pathways in primary astrocytes [29]. Here, we used selective inhibitors of these kinases to determine whether activation of these kinases contributes to the effect of SAA on astrocyte migration and polarization. For Boyden chamber assay and scratch-wound assay, pre-treatment with SB203580 (a p38 inhibitor) significantly attenuated the inhibition of SAA on the migration of U251 cells (Fig. 7a–d; Additional file 1: Figure S2). However, pre-treatment with FR180204 (an ERK1/2 inhibitor), SP600125 (a JNK inhibitor), or LY294002 (a PI3K inhibitor) has no effect on SAA-induced inhibition on cell migration (Fig. 7a–d; Additional file 1: Figure S2). In addition, MTT and Boyden chamber assay showed that SB203580 (10 μM), FR180204 (5 μM), SP600125 (5 μM), or LY294002 (5 μM) incubation for 12 h and/or 16 h had no effect on the viability and migration of U251 cells, respectively (Additional file 1: Figure S1B, C, and S3). These results suggest that p38 MAPK is critically involved in the inhibition of SAA on astrocyte migration.

Next, we further verified whether p38 participates in the effect of SAA on astrocyte polarization. We found that pre-treatment with SB203580 significantly attenuated SAA-induced inhibition of microtubule reorganization in U251 cells and the decrease of protruding cells at the wound edge (Fig. 7e, f). All these data suggest that SAA inhibits astrocyte migration and polarization through activating the p38 MAPK signaling pathway.

## Discussion

Activation of astrocytes is a common pathological hallmark of several CNS diseases. At the early stages of AD pathology, activated astrocytes have been observed to accumulate around senile plaques. Activated astrocytes could reduce Aβ accumulation through phagocytosis, thereby controlling early AD pathology. However, given that chronic over-activated astrocytes participate in Aβ metabolism by interacting with plaques and continuously release pro-inflammatory factors to accelerate the progress of AD, limiting their recruitment to Aβ may also help to inhibit or delay the progression of AD [38]. The present study demonstrates for the first time that an endogenous protein, SAA, is significantly induced in the brain of AD mice and is involved in inhibiting the migration of astrocytes to Aβ plaques. Further mechanistic studies have shown that SAA inhibits astrocyte migration and polarization via disrupting actin and microtubule reorganization and Golgi reorientation. Inhibition of the p38 MAPK pathway abolishes the suppression of SAA on astrocyte migration and polarization. These findings provide new insights into the role of SAA in AD and indicate that SAA expressed in the brain may be a potential target in AD prevention and therapy. In addition, we have also found that SAA deficiency further enhances the activation of astrocytes in the brain of AD mice, suggesting the inhibitory effect of SAA on astrocyte activation in vivo. Several studies have shown that the over-activation of astrocytes participates not only in the pathological process of AD [39], but also in other brain diseases, such as Huntington disease, ischemic stroke, and epilepsy [40]. Our results suggest that SAA may also play a role in the pathological process of these brain diseases.

In this study, the APP/PS1 transgenic mice were used, which have been widely employed in studies of AD. We found that Saa3 expression is significantly induced in the cortex and hippocampus in APP/PS1 mice aged 12 months, and increased Saa3 is mainly distributed in the neurons. Our previous study has found that in a LPS injection model of systemic inflammation, a significant amount of the induced Saa3 protein is in the neurons and, to a much lesser extent, astrocytes in the mouse brain [26]. Murine *Saa3* encodes a functional SAA protein, which is the major form of SAA in inflammatory and extrahepatic tissues [23, 41]. Among the *SAA* functional genes in humans, *SAA1* is the most similar in structure and function to murine *Saa3*, suggesting that SAA1 and Saa3 could be ortholog proteins [17, 42, 43]. It is worth exploring whether and how human SAA1 locally produced in the brain participates in the pathology of AD in the future. In addition, other studies have also reported that SAA is expressed and secreted in glioblastoma (grade IV, most serious astrocytoma) [44] and in the brain after traumatic brain injury [45]. All these findings indicate that the local production of SAA may be related to the pathogenesis of these diseases.

SAA has cytokine-like properties including pro-inflammatory and anti-inflammatory functions in several different cell types [10–16]. Compared with the cytokine-inducing ability of SAA, its chemotactic ability is relatively poorly understood [46]. SAA has been found to simulate the directed migration of monocytes, neutrophils, dendritic cells, endothelial cells, and synovial fibroblasts [10, 14, 46–48]. Here, we clarified that SAA inhibits the directional migration of astrocytes in vivo using APP/PS1-*Saa3*^*−*/*−*^ mice and in vitro via stimulation of primary cultures of mouse astrocytes and human glioma U251 cells with recombinant human apo-SAA. Although the inhibition of SAA on cell migration is rarely reported, Knebel et al. found the different effects of SAA on the migration of two human glioma cell lines. In T98G cells, SAA treatment increased the migration and invasion behaviors, whereas in A172 cells, it decreased these behaviors [49]. These dual effects may be related to the inhibition activity of SAA on MMP-2 and MMP-9 in A172 cells while increasing them in T98G cells [49]. In our study, the inhibition of SAA on astrocyte migration is through the activation of the p38 MAPK pathway. Other studies have also reported that the non-steroidal anti-inflammatory drugs and piperlongumine (a primary constituent of *Piper longum*) inhibit the migration of prostate cancer cells and glioblastoma cells (LN229), respectively, via activation of the p38 MAPKs pathway [50, 51]. However, studies have also found that activating p38 promotes cell migration. In the ubiquitin ligase HERC1-depleted cells, for example, activation of p38 increases the migration of human osteosarcoma U2OS cells [52]. These findings suggest that various stimulants affect cell migration through specific signaling pathways. The same stimulant, such as SAA, may have different effects on the migration of different types of cells, which may regulate their migration through different signaling pathways.

Cell polarization is a requirement of cellular movement and migration. It is characterized by the change in the morphology of the cells at the wound or chemoattractants, the reorganization of actin filaments and microtubules, and the reorientation of the centrosome and the Golgi apparatus to face the direction of migration [32, 53]. In this study, we observed that SAA disturbs actin and microtubule reorganization and Golgi reorientation. Our previous study has shown that SAA suppresses astrocyte proliferation by regulating the cell cycle. Primary cultures of astrocytes display cell cycle arrest in the G1 phase after SAA treatment [29]. Studies have found that microtubules and actin filaments play important roles in mitosis [54]. These cytoskeletal filaments are the targets of a number of anti-cancer drugs. The drugs inhibit cell proliferation by disturbing the polymerization and dynamics of microtubules and actin filaments. Therefore, our results suggest that SAA may inhibit astrocyte proliferation by interfering with the stability of microtubules and actin filaments at the G1 checkpoint, indicating that SAA may also be a potential target for cancer treatment. In addition, cell migration requires the participation and cooperation of a number of extracellular and intracellular signal molecules [37]. Future studies will be required to illuminate whether and how other molecules participate in the regulation of SAA on astrocyte migration.

## Conclusions

The present study demonstrates that SAA plays a role in regulating astrocyte migration. SAA inhibits astrocyte activation and migration to Aβ plaques in APP/PS1 mice, as evidenced by increased activation of astrocytes and the number of astrocytes around Aβ plaques in APP/PS1-*Saa3*^*−*/*−*^ mice. Further mechanistic studies have shown that SAA inhibits astrocyte migration and polarization via disrupting actin and microtubule reorganization and Golgi reorientation. Inhibition of the p38 MAPK pathway serves to the suppression of SAA on astrocyte migration and polarization. Our findings suggest that SAA is a potential target in AD prevention and therapy.

## Supplementary information

**Additional file 1.** Supplementary methods and figures.

## Data Availability

All datasets generated or analyzed during this study are available from the corresponding author on reasonable request.

## Abbreviations

AD: Alzheimer’s disease
Aβ: Amyloid beta
DAPI: 4,6-Diamidino-2-phenylindole
DMEM: Dulbecco’s modified Eagle’s medium
GAPDH: Glyceraldehyde-3-phosphate dehydrogenase
GFAP: Glial fibrillary acidic protein
MAPK: Mitogen-activated protein kinase
MMP: Matrix metallopeptidase
PI3K: Phosphoinositide 3-kinase
SAA: Serum amyloid A
TBS: Tris-buffered saline
WT: Wild type
