# Supplementary material for: Serum amyloid A inhibits astrocyte migration via activating p38 MAPK
Source: J Neuroinflammation. 2020 Aug 29;17:254. doi: 10.1186/s12974-020-01924-z (PMC7456509; doi:10.1186/s12974-020-01924-z)

**Additional file 1.**

**Supplementary Materials**

**Supplementary Methods**

**Cell viability measurement**

Cell viability was measured by a colorimetric assay with methyl thiazolyl tetrazolium (MTT). Primary cultures of astrocytes or U251 cells grown in 96-well plates were exposed to SAA (1 μM), SB203580 (5-10 μM), FR180204 (5-10 μM), SP600125 (5-10 μM), or LY294002 (5-10 μM) for 12 h and 16 h. The culture medium was removed and the cells were incubated with MTT (1 mg/ml) at 37 °C for 4 h. After three washes with phosphate-buffered saline (PBS, pH 7.4), the insoluble formazan product was dissolved in dimethyl sulfoxide. The optical density at 570 nm was determined on a FlexStation III plate reader (Molecular Devices, Mountain View, CA). Cell viability was expressed as a percentage of control.

**Supplementary Figures**

**Figure Legends**

**Figure S1.** Effect of SAA, MAPKs and PI3K inhibitors on cell viability of primary astrocytes and/or U251 cells. (**A**) Primary cultures of astrocytes and U251 cells were exposed to SAA (1 μM), and the cell viability was detected after 16 h by MTT. U251 cells were exposed to SB203580 (5-10 μM), FR180204 (5-10 μM), SP600125 (5-10 μM), or LY294002 (5-10 μM), and the cell viability was detected after 12 h (**B**) or 16 h (**C**) by MTT. Data are expressed as percentage of surviving cells over control cells. Results are expressed as the mean ± SEM based on three independent experiments, each in triplicate. *** *p* < 0.001 compared with the control (DMEM medium).

**Figure S2.** Inhibiting p38 alleviates the interference of SAA on astrocyte migration in a dose-dependent manner. U251 cells were incubated with SAA (1 μM) with or without a 15 min pretreatment with SB203580 (1-10 μM), FR180204 (0.1-5 μM), SP600125 (0.1-5 μM), or LY294002 (0.1-5 μM). At 12 h after incubation, the cell migration was examined by 48-well chemotaxis chambers. Representative images of migrated cells on membrane filters were shown in (**A**) and quantified data were shown in (**B**). Magnification, × 400. Results are expressed as the mean ± SEM based on three independent experiments, each in triplicate. *** *p* < 0.001 compared with the control (DMEM medium). **^#^** *p* < 0.05 compared with SAA treatment group.

**Figure S3.** Effect of MAPKs and PI3K inhibitors on the migration of U251 cells. U251 cells were treated for 12 h with SB203580 (10 μM), FR180204 (5 μM), SP600125 (5 μM), or LY294002 (5 μM). The cell migration was examined by 48-well chemotaxis chambers. Representative images of migrated cells on membrane filters were shown in (**A**) and quantified data were shown in (**B**). Magnification, × 400. Results are expressed as the mean ± SEM based on three independent experiments, each in triplicate.

**Figure S1**


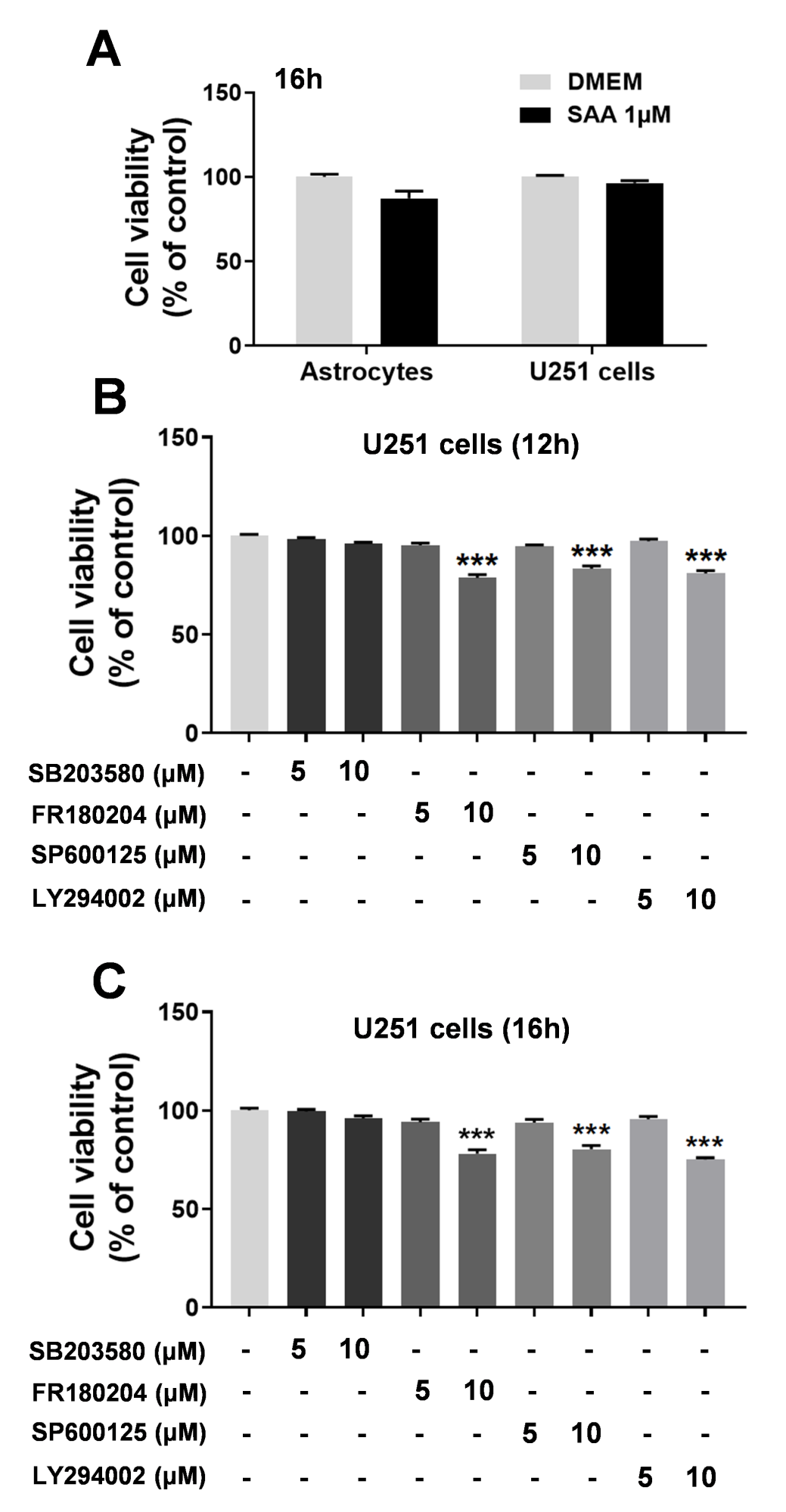


**Figure S2**


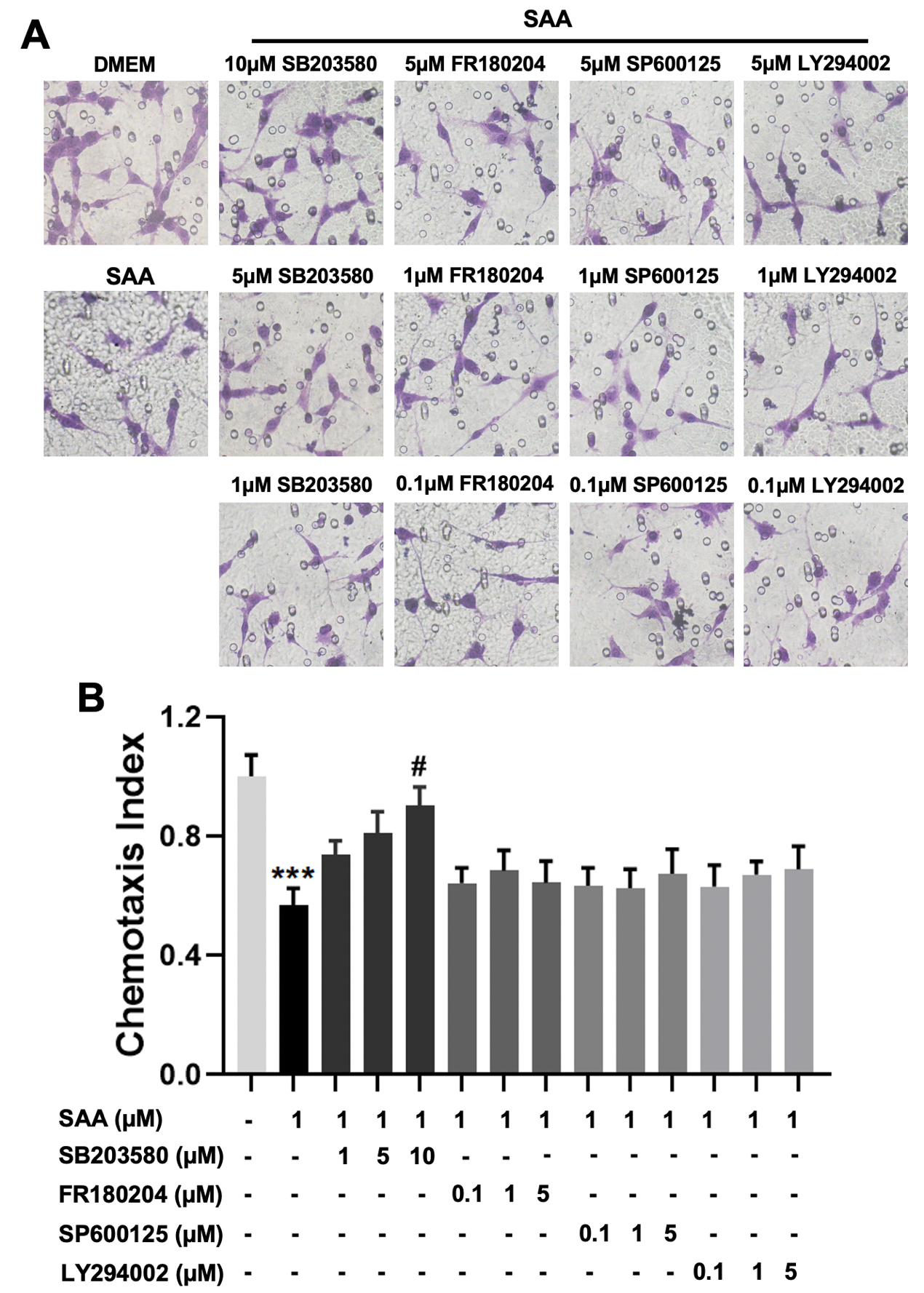


**Figure S3**


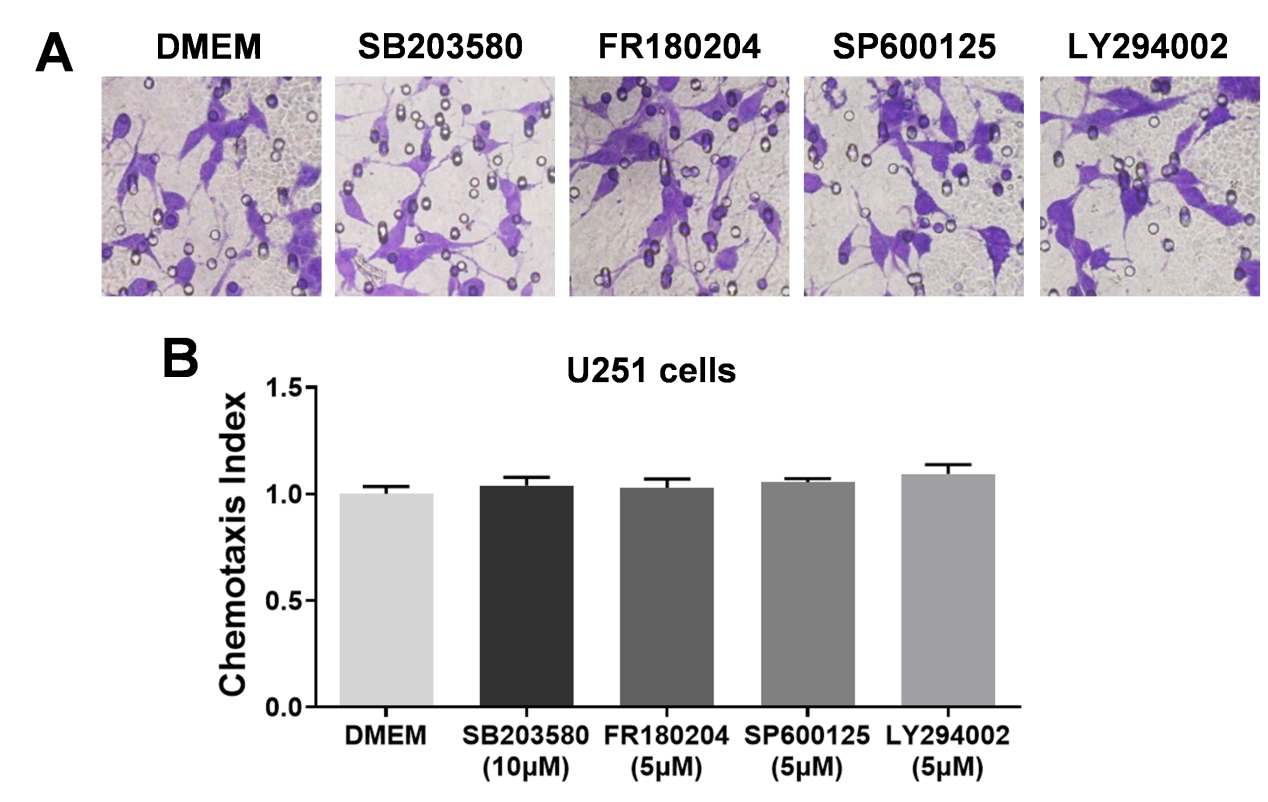

Supplement: Supplementary file 1 — Additional file 1. Supplementary methods and figures. [file 12974_2020_1924_MOESM1_ESM.docx]
